# Luis M. Botana, M. Carmen Louzao and Natalia Vilariῆo (Eds.): Climate change and marine and freshwater toxins

**DOI:** 10.1007/s00216-015-9292-z

**Published:** 2016-01-15

**Authors:** Tamim Younos

**Affiliations:** Green Water-Infrastructure Academy, Washington, DC 20001 USA

**Bibliography:**
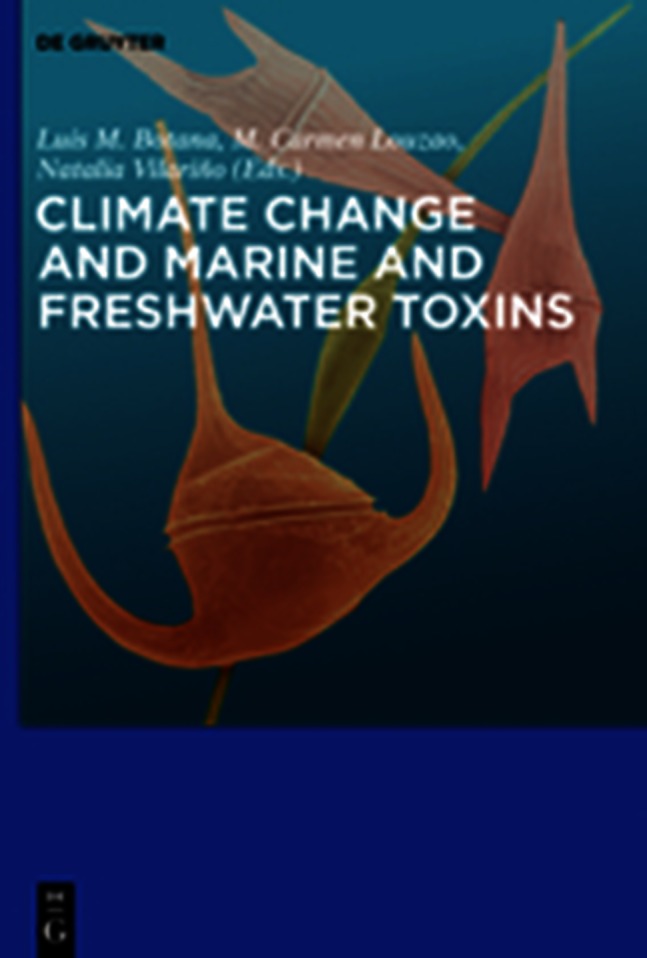
Climate change and marine and freshwater toxins Luis M. Botana, M. Carmen Louzao and Natalia Vilariῆo (Eds.) De Gruyter ISBN: 978-3-11-033303-9 Hardcover, 490 pages August 28, 2015, € 199.95/$ 280.00/£ 149.99

**Book’s topic** How climate change affects the Earth’s natural environment and its consequences for human and animal health are critical issues. While the impact of climate change on weather patterns and its consequences are broadly discussed in the literature, less is known about the possible effects of climate change on water, food, and health. This book focuses on the potential impacts of climate change on toxins in freshwater and marine waters, and offers evidence of complex interactions involving organisms ranging from primary toxin producers right up the food chain to humans.

**Contents** The 13 chapters of this book cover a broad spectrum of topics. Chapter 1 discusses the variability and trends in global sea ice cover and sea level, and their potential effects on physicochemical parameters. Chapter 2 presents new techniques in environmental monitoring. Topics discussed include in-situ harmful algal bloom monitoring, liquid chromatography and mass spectrometry, biosensors for HAB monitoring, advances in nanotechnology for HAB detection, and molecular biology-based techniques for HAB detection. Chapter 3 discusses responses of marine animals to ocean acidification, causes of acidification, and the effects that acid–base disturbances in marine environments can have on animals. Chapter 4 reviews *Alexandrium* spp.: saxitoxins are defined, and genetic and ecological factors that influence saxitoxin production and proliferation are described, as are the taxonomy and species evolution of *Alexandrium* as well as ecological and environmental factors that influence the proliferation of *Alexandrium* spp. Chapter 5 discusses potential effects of climate change on cyanobacterial toxin production, defines cyanobacteria, and explores the effects of climate change on common toxin-producing species and toxin regulation. Chapter 6 focuses on the relationship between climate change and harmful marine algal blooms, algal bloom range extensions, and the challenge of predicting phytoplankton community responses to climate change. Chapter 7 considers the effects of global warming on inland water bodies, the ecology of cyanobacteria and toxin production, and direct and indirect effects of global warming on cyanobacteria growth and microcystin concentration. Chapter 8 discusses emerging toxins in Mediterranean coastal ecosystems, including a discussion of socioeconomic implications of climate change. Chapter 9 scrutinizes the genus *Gambierdiscus*—the cause of fish poisoning (an increasing threat to human health), its geographic distribution and abundance, and the toxicities of different *Gambierdiscus* species in seafood. Chapter 10 discusses the control of harmful algal blooms, such as the management of cyanobacteria in waste stabilization ponds, the treatment of cyanobacteria and cyanotoxins with hydrogen peroxide, and new techniques for the control and characterization of cyanobacterial blooms. Chapter 11 explores the global climate change profile and its possible effects on the reproductive cycle, sex expression, and sex change of shellfish as marine toxin vectors. Chapter 12 discusses various effects of climate change such as foodborne/waterborne diseases and zoonosis and other animal diseases, harmful algal blooms, and microalgal toxicity on world food production and security. Chapter 13 examines the connection between food science and policy, focusing on current worldwide regulations for marine phycotoxins and limitations on the development and implementation of new regulations. It introduces an integrative example of tetrodotoxin as a biomarker of climate change.

**Comparison with the existing literature**

There is scattered scientific information related to the theme of this book, but it has not previously been compiled in a volume that could be used as a comprehensive reference material by researchers. This book is an excellent follow-up to* Seafood and freshwater toxins: pharmacology, physiology, and detection* (edited by Luis M. Botana, CRC Press 2014). Other recently published books on related topics include* Pharmacokinetics and toxicokinetics* (Mehdi Boroujerdi, CRC Press 2015) and* Nutritional freshwater life* (Ramasamy Santhanam, CRC Press 2015).

**Critical assessment**

The chapters of this book could have been organized into a better sequence of topics to avoid repetition. However, the book covers an evolving area of science where scientific evidence may not always be available to prove or disprove the link between climate change and toxins in water and its effects. The 2357 references listed in the 13 chapters provide an excellent guide to the broad spectrum of researchers in this field.

**Readership recommendation**

The book is an excellent introduction to the complex topic of climate change and toxins in water. It is a useful resource for researchers working in environmental monitoring, ecotoxicology, and related fields.

**Summary**

This 13-chapter book focuses on an evolving area of research—the potential impacts of climate change on toxins in freshwater and marine waters, and provides significant evidence of the complex interactions involving organisms ranging from primary toxin producers right up the food chain to humans. The book can serve as useful guide for researchers working in environmental monitoring, ecotoxicology, and related fields.

